# Sex steroid hormone function in the brain niche: Implications for brain metastatic colonization and progression

**DOI:** 10.1002/cnr2.1241

**Published:** 2020-03-03

**Authors:** María J. Contreras‐Zárate, Diana M. Cittelly

**Affiliations:** ^1^ Department of Pathology University of Colorado Denver, Aurora, Colorado

**Keywords:** androgen, androgen‐receptor, aromatase, astrocytes, brain endothelium, estrogen, estrogen‐receptor, microglia

## Abstract

**Background:**

While sex hormones and their receptors play well‐known roles in progression of primary tumors through direct action on sex steroid hormone‐responsive cancer cells, emerging evidence suggest that hormones also play important roles in metastatic progression by modulating the tumor microenvironment. Estrogens and androgens synthesized in gonads and within the brain influence memory, behavior, and outcomes of brain pathologies. Yet, their impact on brain metastatic colonization and progression is just beginning to be explored.

**Recent findings:**

Estradiol and testosterone cross the blood‐brain barrier and are synthesized de novo in astrocytes and other cells within the adult brain. Circulating and brain‐synthesized estrogens have been shown to promote brain metastatic colonization of tumors lacking estrogen receptors (ERs), through mechanisms involving the upregulation of growth factors and neurotrophins in ER+ reactive astrocytes. In this review, we discuss additional mechanisms by which hormones may influence brain metastases, through modulation of brain endothelial cells, astrocytes, and microglia.

**Conclusion:**

A greater understanding of hormone‐brain‐tumor interactions may shed further light on the mechanisms underlying the adaptation of cancer cells to the brain niche, and provide therapeutic alternatives modulating the brain metastatic niche.

## 1 INTRODUCTION

In the past decade, major advances have been made in understanding the cellular and molecular mechanisms that regulate brain metastasis (reviewed in 1). Specific interactions within the tumor microenvironment (TME) have emerged as key to the ability of cancer cells to colonize distant sites, including the brain.^2^, ^3^, ^4^, ^5^, ^6^ While some of the interactions between cancer cells and the microenvironment are common between primary tumors and metastases to various organs, cancer cells that reach the brain encounter a more isolated, highly reactive microenvironment protected by the blood‐brain barrier (BBB), which blocks entry of many circulating molecules and cells found in other organs.^7^ It is now known that brain metastatic colonization is a relatively rare event in which circulating tumor cells are arrested at brain capillaries, extravasate into the brain parenchyma, survive anti‐tumorigenic effects of brain immune‐surveillance cells, including microglia and astrocytes, and colonize the brain niche by growing around existing vessels and adapting to the unique brain microenvironment.^3^, ^8^, ^9^, ^10^, ^11^ Interaction of cancer cells with brain endothelial cells, glial cells, and pericytes are critical for the initial steps of metastatic colonization as well as outgrowth to large macrometastases, in a process that spans months to years from primary tumor diagnosis.

It is well recognized that sex hormones influence both normal and pathological brain functions (reviewed in 12, 13, 14, 15), but until recently, how the hormonal milieu could influence brain metastases remained unexplored. While both males and females develop brain metastases, age and sex influence the metastatic incidence within specific tumor types. For example, lung brain metastases occur with similar frequency between men and women (18.9% vs 21.8%, respectively), while melanoma brain metastases occur more frequently in men than women (8.7% vs 4.8%, respectively).^16^ Younger age and female sex were reported as significant risk factors for subsequent brain metastasis in a subset of advanced non‐small cell lung cancer cases.^17^ In predominantly female breast cancer, young age is a predictive factor for the development of brain metastasis independent of tumor subtype, suggesting age‐dependent and host‐specific factors promote metastases in younger women.^18^, ^19^, ^20^

Although the function of sex hormones (estrogens and androgens) in cancer progression has been best defined in terms of their pro‐tumorigenic function on sex hormone‐receptor expressing cancer cells (see key reviews on this topic^21^, ^22^, ^23^, ^24^, ^25^), their roles in the brain are more varied and complex. Sex hormones act throughout the entire brain of both males and females modulating many cellular and molecular processes, which in turn alter the structure and function of specific brain compartments. In this review, we will discuss the known roles of estrogens and androgens in brain function, and how they may impact brain metastasis through their action on key components of TME.

### 1.1 Peripheral synthesis of sex steroid hormones

The predominant circulating sex steroid hormones after puberty are estrogens in females and testosterone in males. In both sexes, the gonads and adrenal glands synthesize estrogens and androgens and release them into circulation. In females, three major forms of physiological estrogens are present: estrone (E1), estradiol (E2), and estriol (E3). Before menopause, E2 is the most potent circulating estrogen, while E1 is more important after menopause. E3 is the least potent estrogen, though it plays a larger role during pregnancy when it is produced in large quantities by the placenta.^26^

In premenopausal women estrogens are synthetized from cholesterol, mainly in the ovaries and during pregnancy by the placenta, acting as an endocrine factor to maintain ovulation and reproductive capability (reviewed in 27, 28, 29). The level of circulating estrogens depends upon the reproductive status of the individual and is highest during the reproductive years^30^ (Table 1). At menopause, circulating estrogen levels undergo a sustained drop, but androgen production experiences only a small, gradual decline by the ovaries and adrenal glands. After menopause, the ovaries maintain secretion of testosterone and androstenedione, which are converted to E2 and E1 in the breast and other tissues by aromatase enzyme (CYP19).^33^

**TABLE 1:** Circulating levels of sex hormones in adults

Hormone		Female levels	Male levels
E1	Early follicular: Mid cycle: Luteal: Postmenopausal	190.13 (1.85‐761.99) pM^31^ 290.37 (13.21‐1908.68) pM^31^ 314.05 (33.03‐1413.02) pM 140.2 ± 51 pm*^32^ 91.7 (48.8‐164.2) pM^33^	43‐464 PM^34^
E2	Early follicular: Mid cycle: Luteal: Postmenopausal:	194.20 (5.51‐2301.72) pM^31^ 453.37 (5.51‐3582.9) pM^31^ 466.22 (5.51‐1997.02) pM^31^ 2.5 ± 8.9 pm*^32^ 19.5 (9.9‐40.4) pM^33^	29‐197 pm ^34^
T	Early follicular: Mid cycle: Luteal: Postmenopausal:	0.32 (0.04‐0.85) pM^31^ 0.35 (0.09‐1.01) pM^31^ 314.05 (33.03‐1413.02) pM^31^ 721.2 (377.9‐1362.6) pM^33^	^&^17 680 ± 5500 pm*^35^

*Note*: To facilitate comparisons, values originally reported as pg/mL, ng/mL, and ng/dL were converted to SI units (pM) using the following molecular weights: E1, 207.366 g/mol; E2, 272.29 g/mol; and Testosterone, 288.42 g/mol. *Values are mean ± SD. All others are median (range).

In males, the much greater levels of circulating testosterone produced by the mature testes generate and maintain the sexual phenotype (Table 1). However, testes also produce about ~20% of circulating estrogens, with the remainder from local production by adipose tissue, brain, skin, and bone, through conversion of testosterone to estrogen by aromatase.^34^, ^36^, ^37^ Estrogens have thus emerged as the active factors in mediating many of testosterone's effects on target tissues in adult males.^37^, ^38^

While peripheral sex hormone synthesis is generally well‐regulated in healthy individuals, cytotoxic drugs and radiation therapies used to treat systemic and brain metastatic tumors can derail these processes in some patients. When radiotherapy is used to treat brain metastasis, inclusion of the hypothalamic‐pituitary axis in the radiation fields can lead to neuroendocrine dysfunction. In a longitudinal trial of patients with brain gliomas, half of premenopausal women at study entry developed premature menopause, and 37% of men aged less than 50 years had low levels of testosterone.^39^ Similarly, cytotoxic drug treatments in premenopausal women can induce premature ovarian failure, with consequently altered synthesis of ovarian E2.^40^ Thus, sex hormone function alterations as a result of brain metastasis treatments could also play a role in the progression of brain metastasis.

## 2 CENTRAL SYNTHESIS OF SEX‐STEROID HORMONES IN THE ADULT BRAIN

**Figure 1:**
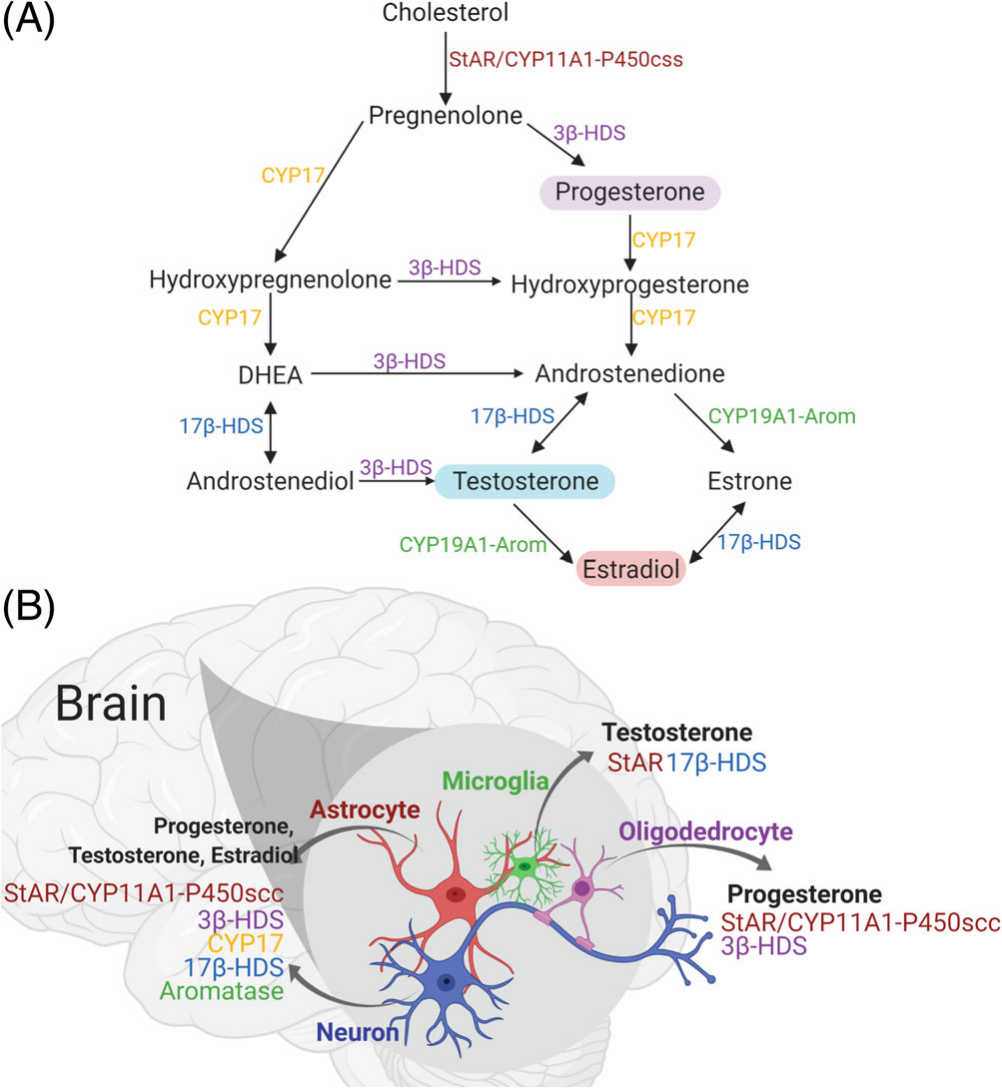
Steroid hormone synthesis by the mammalian brain. Steroid hormones are derived from cholesterol, and several cells within the brain niche possess all the enzymes required to synthesize sex‐hormones de novo. Astrocytes and neuron express all enzymatic machinery StAR (Steroidogenic acute regulatory protein); CYP11A1 or P450scc (Cholesterol side‐chain cleavage enzyme); 3β‐HSD (3‐beta‐hydroxysteroid dehydrogenase); CYP17 (17‐alpha‐hydroxylase/17,20 lyase) and 17β‐HDS (17β‐Hydroxysteroid dehydrogenase) and aromatase to produce progesterone, testosterone, and E2 from cholesterol. Microglia express StAR and 17β‐HDS and are able to synthetize testosterone and androstenodiol from androgenic C19‐steroids. Oligodendrocytes express StAR /CYP11A1 and 3β‐HSD to produce progesterone in the brain^47^, ^49^, ^50^, ^51^, ^52^, ^53^

Because of their lipophilic natures, estradiol and testosterone can cross the BBB. However, the movement of these hormones across the BBB is thought to reflect the combined effects of their lipid solubility and the presence of circulating binding proteins such as albumin or sex‐hormone binding globulins.^41^, ^42^, ^43^, ^44^, ^45^ Therefore, the levels of circulating hormones may not reflect local availability and function of sex hormones in the brain. Korneyev et al demonstrated that pregnenolone, the first steroid formed by mitochondrial oxidative cleavage of cholesterol, is synthesized in the forebrain, cerebellum, and olfactory bulb of adrenalectomized and castrated Sprague‐Dawley male rats treated with trilostane (an inhibitor of pregnenolone metabolism from progesterone), demonstrating the functionality of brain cytochrome P450 side‐chain cleavage (CYP11A1), as well as the ability of the brain to produce sex hormones *de novo*.^46^ In the brain, astrocytes are the most active steroidogenic cells (at least in murine models), expressing CYP11A1, 17alpha‐hydroxylase/C17‐20‐lyase (CYP17), 3beta‐hydroxysteroid dehydrogenase (3β‐HSD), 17beta‐hydroxysteroid dehydrogenase (17β‐HSD), and cytochrome P450 aromatase (CYP19A1).^47^ Astrocytes have been shown to produce pregnenolone, progesterone, dehydroepiandrosterone (DHEA), androstenedione, testosterone, E2, and E1. Oligodendrocytes express only CYP11A1 and 3β‐HSD to produce pregnenolone, progesterone, and androstenedione, but lack the enzymes necessary to produce DHEA, testosterone, or estrogens. Neurons express CYP11A1, CYP17, 3β‐HSD, and CYP19A1 to produce pregnenolone, DHEA, androstenedione, and estradiol, but do not express 17β‐HSD or produce testosterone^47^, ^48^ (Figure 1).

Brain E2 biosynthesis from androgenic precursors (testosterone and androstenedione) by the aromatase enzyme has been recognized for several years.^54^, ^55^, ^56^ Early studies using ^3^H‐testosterone demonstrated aromatase activity in homogenates of hypothalamus,^57^ amygdala, and hippocampus^58^ from human fetuses, and also other mammals.^59^, ^60^, ^61^, ^62^, ^63^, ^64^ However, brain aromatase expression is regulated in a species‐ and region‐selective manner. In humans, the regional distribution pattern of aromatase was found to be strikingly different from the distribution reported in non‐human primates (baboon and rhesus monkey) and rodents.^65^, ^66^ Biegon et al used a radiolabeled aromatase ligand ([^11^C]vorozole) alone or in combination with the aromatase inhibitor letrozole to trace regional aromatase distribution by positron emission tomography, they reported a highly heterogeneous distribution of aromatase activity, with the highest levels in the thalamic nuclei, followed by moderately high levels in amygdala, preoptic area (POA), and medulla and low levels in cortex, putamen, cerebellum, and cortical white matter.^65^ Premenopausal women showed higher aromatase activity than postmenopausal women but brain uptake of C‐vorozole did not vary across the menstrual cycle in pre‐menopausal women,^66^ suggesting that brain E2 synthesis (or at least aromatase function) is not regulated by circulating levels of E2. Males showed increased brain aromatase activity compared to healthy females, supporting the notion that brain estrogens mediate brain‐functions in males and females. Given that testosterone can also be reduced to dihydrotestosterone in the brain (Figure 1), and that dihydrotestosterone is a more potent androgen than testosterone,^15^ regulation of aromatase and 17β‐HSD is likely to play key roles in defining the ultimate action of sex hormones in the male and female brain.

Neurons, astrocytes, and endothelial cells from rodents and humans express aromatase.^38^, ^67^, ^68^, ^69^, ^70^, ^71^, ^72^ Increased brain aromatase expression or activity have been demonstrated following hypoxia and ischemia,^73^, ^74^ increased pressure,^68^ and mechanically or chemically induced brain injuries in male and female rodents.^75^ Similarly, aromatase upregulation appears to be a response to reactive gliosis initiated by brain metastases, as aromatase can be detected in reactive astrocytes from resected breast cancer brain metastasis (unpublished data). Recent studies showed that ovariectomy combined with the aromatase inhibitor Letrozole was more effective in blocking brain metastatic colonization in an experimental model of estrogen‐unresponsive (triple negative) breast cancer brain metastasis, compared to ovariectomy alone.^76^, ^77^ This provided the first pre‐clinical evidence that both ovarian and peripheral E2 contribute to brain metastatic colonization by acting on the brain microenvironment rather than the tumor itself. However, whether aromatase inhibition could decrease metastatic colonization by other primary tumors, and whether this function is similar in males and females, remains unclear.

## 3 SEX HORMONE SIGNALING IN BRAIN METASTASIS: DIRECT ACTION ON CANCER CELLS

**Figure 2:**
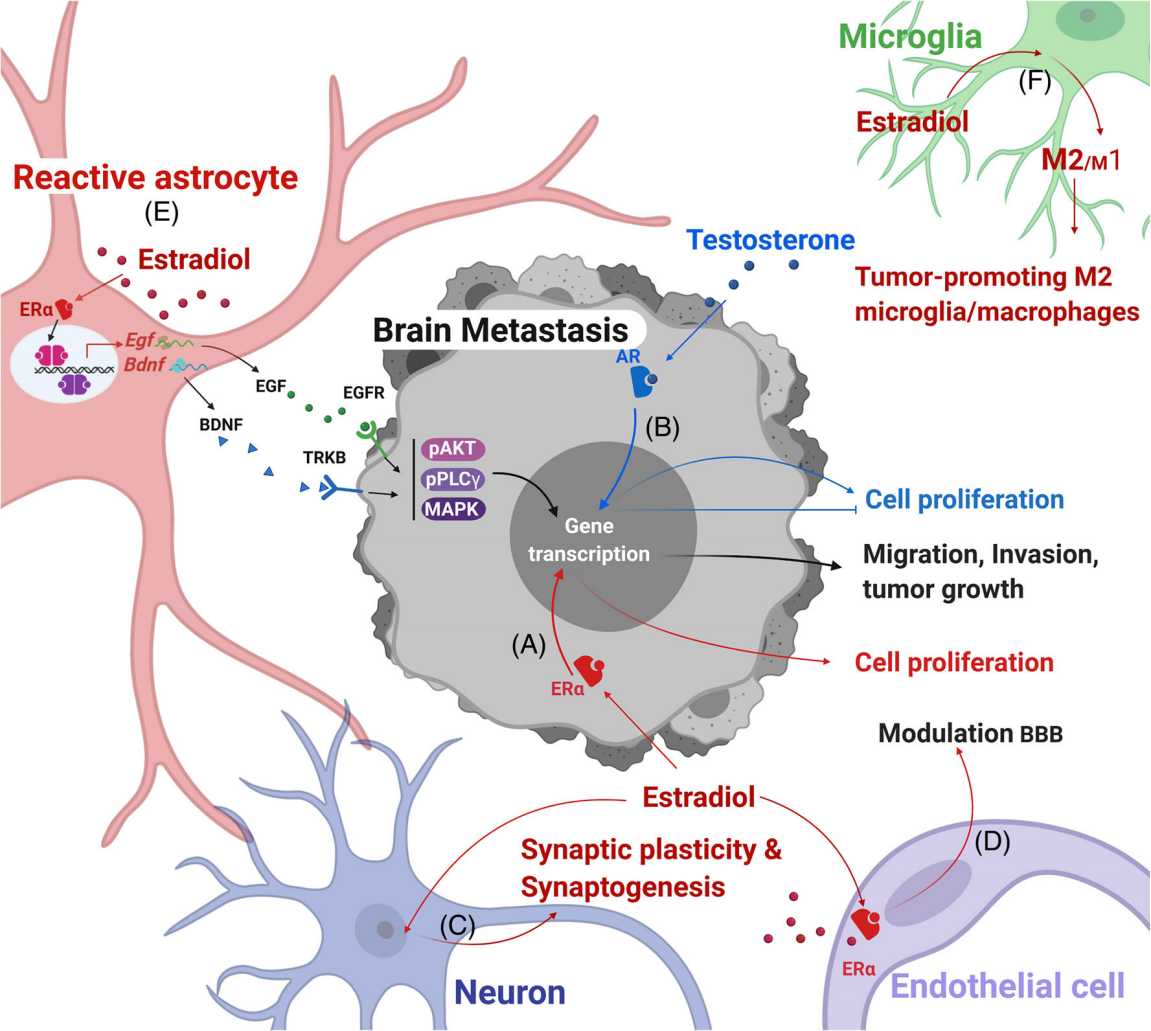
Mechanisms by which sex hormones may influence brain metastasis. A, Direct action of local estrogens is likely to influence proliferation of ER+ breast cancer brain metastasis. B, Direct action of androgens on AR+ brain metastasis can have proliferative and anti‐proliferative effects in tumor cells. C, Estrogen may influence the ability of metastatic cancer cells to form pseudosynapses with neurons. D, Estrogens can modulate BBB permeability by regulating endothelial tight junctions and pro‐angiogenic factors (VEGF, HIF). E, E2 acts through ER+ reactive astrocyte to increase secretion of growth factors that activate canonical oncogenic epidermal growth factor receptor (EGFR) and TRKB on brain metastatic cancer cells. F, Estradiol may promote microglia polarization and recruitment of immunosuppressive cells to the brain niche

Direct mechanisms of sex hormone function on cancer cells have been extensively described in the context of hormone receptor‐positive (HR+) breast and prostate cancer (reviewed in 24, 78, and 79), and similar mechanisms likely impact HR+ growth in the brain (Figure 2A,B). In this section, we will briefly review mechanisms of direct hormone signaling in cancer cells, followed by evidence linking hormone receptor function to brain metastasis progression of HR+ brain metastasis. In Figure 2, we provide an integrated summary of how sex hormones acting directly on cancer cells and the brain niche may participate in brain metastasis.

Estrogens act via estrogen receptors (ERs, ERα, and ERβ) or through G protein‐coupled ER1 (GPER1, also known as GPR30).^80^, ^81^ Estrogen binding to these receptors initiates classic nuclear‐initiated steroid signaling (NISS, also known as genomic signaling) and membrane‐initiated steroid signaling (MISS, previously known as non‐genomic signaling).^82^ During NISS, E2 binding triggers intracellular localization of ERα and ERβ, which dimerize and enter the nucleus, binding to E2 response elements (EREs), or activator protein‐1 (Ap1) and specificity protein‐1 (Sp1), on the promoter of E2 responsive genes to regulate transcription. In the MISS pathway, E2 binds to membrane‐bound ERα and ERβ receptors as well as GPR30 to rapidly activate nuclear transcription factors via the MAPK pathway.^56^, ^83^, ^84^, ^85^ By contrast, androgens bind to one androgen receptor (AR) and signal through NISS.^86^ The AR is primarily located in the cytoplasm. Following ligand‐binding, AR translocates to the nucleus to bind androgen response elements. This enables recruitment of histone acetyltransferases, coactivators, and other proteins crucial for transcriptional machinery.^87^, ^88^, ^89^ Some studies suggest that MISS can be induced by testosterone; however, whether this occurs via membrane‐bound AR or an unknown receptor remains unclear.^83^, ^90^, ^91^

Estrogens are well known to play a mitogenic role in breast cancer via direct activation of ERα and endocrine therapy (ie, Selective ER Modulators, ER‐antagonists, and aromatase inhibitors) are the main form of targeted therapy for these tumors. The direct role for estrogens on brain metastasis of ER+ breast tumors has received less attention, due to the clinical observations that (a) the incidence of brain metastasis is lowest in patients with ER+ breast tumors compared to breast tumors lacking ER (ie, HER2+ or Triple Negative Breast cancer, TNBC)^92^, ^93^; (b) receptor conversion, particularly loss of hormone receptors, is a common event in brain metastases from breast cancer, and endocrine therapy may increase its incidence^94^, ^95^, ^96^; and (c) metastatic disease in ER+ disease is often a late life event occurring mostly in post‐menopausal women with low levels of circulating estrogens. Yet, around 11% of ER + Her2‐ and 15% of Her2+ ER+ patients with metastatic breast cancer develop brain metastases,^97^ suggesting that extragonadal hormone production in the brain and other organs might drive the slow but eventual development of hormone‐dependent metastases in postmenopausal women. Supporting this hypothesis, a recent study investigating the impact of endocrine therapy after diagnosis of brain metastasis on outcome and clinical course of disease in patients with ER+ metastatic breast cancer, found that continuing endocrine therapy after brain metastasis diagnosis was associated with a significantly prolonged overall survival.^98^ Unfortunately, models of brain metastatic ER+ breast cancer are limited, and ER+ breast cancer cells commonly used to study primary tumor growth in xenograft models require estrogen supplementation to mimic estrogen levels found in humans. Thus, a model of ER+ brain metastasis that grows in a postmenopausal setting common to ER+ breast cancer brain metastasis remains a challenge.

Androgens also play a role in breast, melanoma, and lung cancer primary tumor growth, and may impact their metastasis through similar mechanisms.^24^, ^25^, ^99^, ^100^ AR is expressed in several types of lung cancer, including small cell, adenocarcinoma, and squamous cell carcinoma,^100^ and dihydrotestosterone (DHT) induces a proliferative response in lung cancer cells through cross‐talk of AR and EGFR.^99^ In breast cancer, 60% to 90% of human breast tumors express AR,^101^, ^102^ though biological effects of androgens vary in different breast cancer models,^103^, ^104^, ^105^ with both anti‐proliferative^106^, ^107^, ^108^, ^109^ and proliferative effects reported.^110^, ^111^ However, the extent to which androgen/AR function plays a direct role on brain metastases from breast or other tumors remains to be defined.

## 4 SEX HORMONE SIGNALING IN BRAIN METASTASIS: INDIRECT ACTION ON THE BRAIN NICHE

Androgens regulate a wide array of neural functions, from reproduction to mood and cognitive abilities.^91^, ^112^ In the human brain, nuclear and cytoplasmic AR immunoreactivity has been shown in frozen and paraffin‐embedded sections of the temporal cortex, specifically in neurons, astrocytes, oligodendrocytes, and microglia cells.^113^ In rodents, both male and female brains show strong AR activity in specific regions of the brain including the cerebral cortex, thalamus, and pituitary gland.^114^

Estrogens act via ERα and ERβ and signal through NISS and MISS.^115^, ^116^ ERs are expressed widely in different brain regions and cells, mediating E2‐signaling in a cell type and region specific manner.^117^ The expression of ERα is strongly in brain regions such as the POA, bed nucleus stria terminalis (BNST), amygdala, periventricular nucleus, ventrolateral part of the hypothalamic ventromedial nucleus, and the arcuate nucleus. Likewise, ERβ is found in many of the same regions than ERα but is more highly expressed in the BNST, POA, paraventricular nucleus of the hypothalamus, and supraoptic nuclei, with some variation across species. ERα and ERβ are also found in other brain regions including the hippocampus, midbrain, cortex, diagonal band of Broca, and basal nucleus of Meynert (reviewed in 117). High GPR30 expression has been shown in the hypothalamic‐pituitary axis, hippocampal formation, and brainstem autonomic nuclei.^118^ There is evidence that GPR30 receptors are two to four times more abundant than ERα or ERβ in the prefrontal cortex of the rat.^119^

Expression of specific ERs varies during aging^120^ and also during brain pathology. For example, brain ischemia models demonstrate a twofold to threefold increase in ERα, whereas ERβ expression in neurons decreases.^121^, ^122^ Importantly, ERα, ERβ, and/or GPR30 are expressed in key subcellular compartments within the brain metastatic niche, including neurons, endothelial cells, astrocytes, and microglia.^123^, ^124^ In the next section, we explore the mechanisms by which sex hormones can influence these cell types and their potential impact on brain metastases. Given the difficulty in differentiating between the effects of T vs those of brain‐produced E2, we will focus on the better‐understood roles of estrogen in regulating neuronal function, the BBB, and neuro inflammation that could influence brain metastasis.

### 4.1 Hormonal regulation of neuronal function

Recent studies have demonstrated that brain metastatic growth of breast cancer cells depends on their ability to form synapses with glutamatergic neurons.^125^ Breast cancer lines selected for their proficiency in causing brain metastases showed high expression of phospho‐GluN2B and other subunits of N‐methyl‐D‐aspartate receptor (NMDAR), as well as NMDAR‐mediated currents and calcium transients in response to glutamate or NMDA.^126^ Similar findings in glioma cells^127^, ^128^ suggest that the existence of synaptic structures involving presynaptic neuros and tumor cells post‐synapsis may be common to brain metastasis and primary brain tumors. Hormones could influence this newly recognized “synaptic” interaction of cancer cells with neurons.

For example, estrogen plays important roles in cognitive function, mediated by its ability to rapidly enhance excitatory synaptic transmission, especially via NMDAR‐mediated synaptic activity and long‐term potentiation (LTP).^129^, ^130^, ^131^, ^132^, ^133^ Estrogen promotes the formation of new dendritic spines and excitatory synapses in the hippocampus and cortex^134^ via the activation of membrane ERs,^135^ which subsequently enhance NMDAR transmission and LTP.^136^, ^137^ Acute E2 treatment results in the recruitment of postsynaptic density protein 95 (PSD‐95) to novel dendritic spines, and the NMDA receptor subunit GluN1 is recruited to nascent synapses in cortical neurons.^138^ Androgens and the receptor have also been shown to play a role in remodeling of spine synapsis in limbic brain areas,^139^ suggesting that both estrogens and androgens could influence the ability of cancer cells to form synapses in some brain regions. Future studies should address the extent to which estrogen and potentially androgens modify the ability of cancer cells to form pseudosynapses.

### 4.2 Hormonal regulation of brain endothelial function

Brain endothelial cells are the first cell‐type encountered by disseminated cancer cells at metastatic sites, and studies have shown that cancer cells produce a variety of molecules that destabilize endothelial‐tight junctions and allow extravasation. Brain endothelial cells express ERs and E2 is a well‐known regulator of endothelial cell function,^140^ however, a comprehensive characterization of the effects of E2 on brain endothelial cells and its impact on the blood‐tumor barrier are largely unknown.

E2 has been shown to promote primary tumor progression and metastasis by increasing intratumoral vessel density and improving vessel stabilization to prevent tumor hypoxia and necrosis,^141^ thus it could be predicted that similar pro‐tumorigenic effects take place at the blood‐tumor barrier. Since brain metastases have been shown to grow in close attachment to existing vessels (vessel co‐option), it is likely that estrogenic function in brain endothelial cells plays a role regulating cell migration and intravasation of circulating cancer cells through the brain parenchyma and outgrowth of micrometastases. However, E2 regulates endothelial cells and BBB function in manners that would predict pro‐metastatic and anti‐metastatic effects. For example, E2 induces vasodilatation by increasing nitric oxide synthesis through ERα‐dependent MISS, activation of Pi3K/AKT pathway and GPR30 signaling.^142^, ^143^, ^144^, ^145^ Increased vascular permeability and vasodilation at early stages of brain metastases may promote brain metastasis by increasing tumor blood flow. On the other hand, estrogens decrease BBB permeability by directly regulating expression of tight junction proteins such as claudin 5.^146^ In a mouse model of lipopolysaccharide (LPS)‐induced inflammation, ovariectomy alone did not affect the degree of basal Evans blue dye extravasation, but the presence of LPS (3 mg/kg body weight i.p. assessed 4 hours post‐injection) significantly enhanced paracellular permeability.^147^ Thus, increased E2 levels could predict a decrease in paracellular permeability that would oppose cancer cell extravasation and brain colonization. How paracrine effects of E2 on brain endothelial function may impact brain metastases remains to be addressed experimentally.

### 4.3 Hormonal regulation of neuroinflammation

#### 4.3.1 Direct effects on astrocytes

Interactions of metastatic cells with astrocytes occur at both early and late stages of the colonization process and play pro‐tumorigenic as well as anti‐tumorigenic role. Induction of astrogliosis (activation of astrocytes and microglia) is an early event during metastatic colonization.^148^ Valiente et al proposed that early contacts between tumor cells and astrocytes result in tumor cell death of the majority of tumor cells reaching brain, and only a subset of cells can adapt to avoid this pro‐apoptotic fate.^10^ Both testosterone and estrogens decrease astrogliosis elicited by various brain insults in animal models. For example, early and delayed administration of testosterone or E2 resulted in a significant decrease in the number of vimentin‐immunoreactive astrocytes as well as reactive microglia in a model of brain injury in rodents.^149^ Thus, it is tempting to speculate that increased local levels of estrogens (ie, in brains of younger females and males) or testosterone (in male brains) could suppress early cancer‐cell elicited astrogliosis and favor the survival of disseminated tumor cells.

Accumulating evidence shows that reactive astrocytes switch from a tumor‐suppressive stage to a tumor‐promoting role at later steps of the metastatic cascade and that astrogliosis is exploited by the tumor cells to support their growth.^5^, ^150^, ^151^, ^152^ Reactive astrocytes surrounding human brain metastasis as well as experimental brain metastasis models express ERs, and E2‐treated astrocytes have been shown to activate key pro‐metastatic pathways in cancer cells to promote brain metastatic colonization.^76^, ^77^ For example, E2 was shown to upregulate brain‐derived neurotrophic factor (BDNF) and epidermal growth factor in ER+ astrocytes, which then activate their cognate receptors tropomyosin receptor kinase B (TrkB) and EGFR in breast cancer cells, promoting tumor‐initiating capability, migration and invasion. These studies provided a molecular mechanism by which E2 can influence oncogenic signaling in cancer cells to promote brain colonization. Importantly, testosterone has been shown to increase BDNF expression in hippocampal neurons,^153^ suggesting that upregulation of BDNF by androgens may impact metastatic colonization in males as well. This can be particularly relevant for brain metastasis derived from primary lung cancer, which is often dependent on tyrosine receptor kinase signaling.^154^

It has been shown that the formation of gap junctions between astrocytes and tumor cells through connexin 43 (CX43), allows the passage of cyclic guanosine monophosphate‐adenosine monophosphate, which activates the stimulator of interferon genes (STING) pathway in astrocytes and promotes expression of IFNα and TNFα to further facilitate brain metastatic growth.^155^ Interestingly, hypothalamic CX43 expression is regulated by steroid hormones in a brain region‐specific and sexually dimorphic manner. Estrogen alone or in combination with progesterone significantly increased CX43 protein levels in some regions of the female rat brain, while both hormones significantly decreased CX43 levels in equivalent regions of male rat brains.^156^ Therefore, gap junctional communication with astrocytes can differentially influence brain metastatic colonization in males and females under various hormonal stages.

Immunosuppressive mechanisms are vital to the survival and outgrowth of disseminated cancer cells^157^ and astrocytes play key roles in the modulation of innate and acquired immunity in brain metastases.^3^, ^158^ Consistent with pro and anti‐tumorigenic roles of astrocytes in brain metastatic progression, estrogens can induce pro‐inflammatory (tumor suppressive) and immune‐suppressive (tumor promoting) features in astrocytes. Physiological and pharmacological concentrations of E2 exhibit potent anti‐inflammatory activity in the central nervous system (CNS) by suppressing production of pro‐inflammatory cytokines, such as IL‐6, IL‐1β, and TNFα in many neurological disorders.^159^ However, many studies that address the anti‐inflammatory properties of E2 in the brain and periphery have yielded different results depending on many factors, including the disease model, species, experimental outcome, whether the study is *in vivo* or *in vitro*, estrogenic formulation, and concentration of E2 or other estrogens.^160^, ^161^, ^162^ In the brain, activation of ERα by E2, directly repressed NFκβ‐dependent transcription and suppressed TNFα‐induced NFκB recruitment to the CCL2 enhancer in reactive astrocytes, suppressing astrocytic CCL2 production in a model of experimental allergic encephalomyelitis.^163^, ^164^, ^165^ In contrast, E2 did not attenuate CCL2 levels in a model of LPS‐induced neuroinflammation.^166^ Given that CCL2‐expressing astrocytes mediate the extravasation of T lymphocytes in the brain, and that CCL2 facilitates the process of both migration and infiltration of several cell systems such as monocytes, natural killer cells, T lymphocytes, and memory cells,^167^ defining how E2 modulates astrocytic CCL2 expression during different stages of metastatic colonization may shed light into the specific contribution of E2 to the transition of a tumor‐suppressive early brain niche to a tumor‐promoting immunosuppressive late brain niche.

#### 4.3.2 Direct effects on microglia

Additional to their effects on astrocytes, sex hormones can also directly influence microglia activation and polarization states. Following brain lesions, microglia become active and assume an amoeboid phenotype and a high metabolic rate, synthesizing and secreting several cytokines, such as interleukin IL6, IL1β, and TNFα.^168^, ^169^ E2 also inhibited microglia activation via GRP30 in a model of ischemic stroke.^170^ In ovariectomized rats, low‐dose E2 profoundly suppressed microglia activation and quantitatively shifted microglia from their “activated,” amoeboid morphology to a “resting” state. Further studies using previously defined nomenclature to classify macrophage polarization states into pro‐inflammatory, anti‐tumor M1 macrophages, and immune‐suppressive, tumor‐promoting M2 macrophages, showed that E2 robustly suppressed the “pro‐inflammatory” M1 phenotype, while enhancing the “anti‐inflammatory/immune‐suppressive” M2 microglia phenotype in the hippocampus after global cerebral ischemia.^171^ *In vitro*, E2 acts via estrogen receptor β (ERβ) to enhance the phagocytic clearance of apoptotic cells, and stimulation of either ERβ or GPR30 promoted the adoption of an anti‐inflammatory/immune suppressive phenotype.^172^, ^173^ However, in a model of endotoxin‐induced brain inflammation, expression of genes encoding key cytokines involved in the transfer from the innate to adaptive immunity (TLR2, TNF‐α, and IL‐12) in microglial cells was largely inhibited in the brain of ovariectomized mice at 24 hours post‐challenge, and E2 rescues this effect in an manner dependent on ERα.^174^ Specific studies addressing how hormones alter microglial function in the context of brain metastases are necessary to fully understand their contribution to the pathogenesis of brain metastasis.

#### 4.3.3 Effects on systemic immune infiltration in the brain

Recent studies have shown that E2 plays a pro‐tumorigenic role in primary tumors that lack ERs through driving the mobilization of myeloid‐derived suppressor cells (MDSCs) and enhancing their intrinsic immunosuppressive activity *in vivo*.^175^, ^176^, ^177^ This effect of E2 appears to involve direct as well as paracrine function of E2 on MDSC. Direct E2 binding to ERα activates the STAT3 pathway in human and mouse bone marrow myeloid precursors by enhancing JAK2 and SRC activity,^175^ and directly enhances both the expansion and suppressive activity of M‐MDSCs.^178^ Indirectly, estrogen stimulates cancer‐associated fibroblasts to secrete SDF‐1α, which can recruit tumor‐infiltrating MDSCs to the TME.^179^ Additional to effects on MDSCs, E2 can increase the immune tolerance of tumors by promoting proliferation of the immunosuppressive CD4+ CD25+ Treg phenotype,^180^ and increasing FOXP3 expression in Treg via ERα.^181^ However, there are no reports defining whether similar mechanisms play a role in the context of brain metastasis, particularly in early stages of brain metastatic colonization where the BBB is still intact and traffic of MDSCs to the brain niche might be limited.

## 5 CONCLUDING REMARKS

While the critical role of the brain microenvironment in brain metastases is now well recognized, the complexity of the interactions between cancer cells and specialized cells within the brain niche adds to the challenge of identifying effective and safe therapeutic strategies to target the pro‐metastatic events occurring within the brain niche. Preclinical data using ER‐ breast cancer models in female mice suggest that sex‐hormones, particularly E2, plays a pro‐metastatic function at least in this subset of tumors. While the mechanisms by which E2 promotes metastasis remain to be fully elucidated, a promising therapeutic strategy may involve the use of estrogen‐depletion therapies including aromatase inhibitors, which are already FDA‐approved for the treatment of ER+ breast cancers. Given the pleiotropic roles of estrogens in normal brain functioning, it is critically important to balance the antitumoral benefits of such therapies with the possible mood and cognitive impairments associated with brain estrogen‐deprivation. Additional studies are needed to define whether aromatase inhibition has therapeutic value in males and in brain metastases from lung cancer and melanoma, and to further elucidate the mechanisms by which sex‐hormones alter early and late stages of brain metastatic colonization.

## ETHICAL STATEMENT

Not Applicable.

## CONFLICT OF INTEREST

The authors have stated explicitly that there are no conflicts of interest in connection with this article.

## AUTHOR CONTRIBUTIONS

All authors had full access to the data in the study and take responsibility for the integrity of the data and the accuracy of the data analysis. Conceptualization, M.J.C. and D.C.; Formal Analysis, M.J.C. and D.C; Writing ‐ Original Draft, M.J.C. and D.C; Writing ‐ Review & Editing, M.J.C. and D.C.; Funding Acquisition, M.J.C. and D.C

## Data Availability

Data sharing is not applicable to this article as no new data were created or analyzed in this study.
